# Genome-Wide Association Study of Insertions and Deletions Identified Novel Loci Associated with Milk Production Traits in Dairy Cattle

**DOI:** 10.3390/ani14243556

**Published:** 2024-12-10

**Authors:** Lu Zhao, Jun Teng, Chao Ning, Qin Zhang

**Affiliations:** Shandong Provincial Key Laboratory for Livestock Germplasm Innovation & Utilization, College of Animal Science and Technology, Shandong Agricultural University, Tai’an 271018, China; zl15598321759@163.com (L.Z.); tengjun0520@163.com (J.T.); ningchao@sdau.edu.cn (C.N.)

**Keywords:** INDEL-based GWAS, whole-genome sequence data, milk production traits, dairy cattle

## Abstract

Genome-wide association study (GWAS) have identified a large number of SNPs associated with milk production traits in dairy cattle. Behind SNPs, INDELs are the second most abundant genetic polymorphisms in the genome, which may exhibit an independent association with complex traits in humans and other species. However, there are no reports on GWASs of INDELs for milk production traits in dairy cattle. In this study, using imputed sequence data, we performed INDEL-based and SNP-based GWASs for milk production traits in a Holstein cattle population. We identified 58 unique significant INDELs for one or multiple traits. The majority of these INDELs are in considerable LD with nearby significant SNPs. However, through conditional association analysis, we identified nine INDELs which showed independent associations. Genomic annotations of these INDELs indicated some novel associated genes, i.e., TRNAG-CCC, EPPK1, PPM1K, PTDSS1, and mir-10163, which were not reported in previous SNP-based GWASs. Our findings suggest that INDEL-based GWASs could be valuable complement to SNP-based GWASs for milk production traits.

## Simple Summary

The genome-wide association study (GWAS) is a powerful tool for identifying genomic variants associated with complex traits in dairy cattle. So far, almost all GWASs in dairy cattle have focused on the relationship between SNPs and traits of interest. In addition to SNP-based GWASs, INDEL-based GWASs are also valuable for capturing genetic loci responsible for traits of interest which may not be captured by SNP-based GWASs. In this study, we performed SNP-based and INDEL-based GWASs for milk production traits and identified the INDELs which were independently associated with the traits and genes related to them. Our findings indicate that INDEL-based GWASs could be a valuable complement to SNP-based GWASs for milk production traits.

## 1. Introduction

The genome-wide association study (GWAS) is a powerful tool for identifying genomic variants associated with complex traits, which provide a promising basis for identifying functional genes/loci for the traits. According to statistics, the majority of GWAS research has concentrated on the relationships between SNPs and the desired characteristics. Small insertions and deletions (INDELs), defined as short insertions and deletions (ranging from 1 to 10,000 bp), are the second most abundant genetic polymorphisms in the genome. Mills et al. reported an initial map of INDELs in the human genome that contains 415,436 unique INDELs (one INDEL every 7.2 kb on average) [1]. In the cattle genome, 1000 Bull Genome Project Run 6 identified 1.76 million INDELs along with 42.92 million SNPs [2]. The amount of genetic variation that is caused by these small INDELs is substantial, with similar levels of variation as SNPs. Many of these INDELs map to functionally important sites within genes and, thus, are likely to influence traits and diseases [3,4]. Indeed, INDELs have been found to be highly associated with multiple diseases in humans. Nearly 24% of Mendelian diseases are caused by INDELs [5]. Associations between INDELs and complex diseases in human were also revealed by several INDEL-based GWAS. For example, Hoffmann et al. reported that a single INDEL in the 6q25.3 locus was associated with the risk of prostate cancer [6]. Tao et al. found that a 5 bp INDEL in the GAS5 gene was related to hepatocellular carcinoma risk [7]. Sun et al. reported that a six-nucleotide deletion variant in the CASP8 promoter could reduce the risk of multiple cancers [8]. Dai et al. identified four INDELs which are associated with the susceptibility of lung cancer [9]. In cattle, Ju et al. reported eight INDELs in the *FHIT* gene that were associated with milk traits in Xinjiang brown cattle [10]. Jiang et al. found that 13 INDELs in 11 genes were related to milk composition traits in the Chinese Holstein population [11]. Mesbah-Uddin et al. performed INDEL-based GWASs and identified 178 INDELs in Holstein and 189 INDELs in Nordic Red, which were associated with female fertility traits [12]. These findings indicate that INDELs could be potentially functional genetic variants for complex traits.

With the advancement in genome sequencing techniques, it is now feasible to conduct large-scale genome-wide INDEL genotyping. So far, almost all GWAS studies for milk production traits in dairy cattle have focused on the relationship between SNPs and traits of interest, which revealed a large number of QTLs/associations, as summarized in the cattle QTL Database (CattleQTLdb, https://www.animalgenome.org/cgi-bin/QTLdb/BT/index, accessed on 1 June 2024). On the sequence level, the majority of INDELs are in considerable linkage disequilibrium (LD) with one or multiple SNPs, but there are still some INDELs which are in weak LD (*r*^2^ < 0.1) with or independent to SNPs, as revealed in the above mentioned INDEL-based GWASs. Therefore, in addition to SNP-based GWASs, INDEL-based GWASs are also valuable for capturing genetic loci responsible for traits of interest which may not be captured by SNP-based GWASs. In this study, we aimed to identify INDELs which are independently associated with milk production traits in dairy cattle. Our findings offer a valuable complement to traditional SNP-based studies and provide novel insights into the genetic architecture underlying milk production traits in dairy cattle.

## 2. Materials and Methods

### 2.1. GWAS Population

The population for GWAS consisted of 6649 Holstein cows from 53 dairy cattle farms distributed mainly in Beijing and Shanghai in China. These cows were genotyped with different-version SNP chips, including Illumina Bovine SNP50v1 (50K), Illumina Bovine SNP50v2 (50K), GeneSeek Genomic Profiler Bovine HD (80K), GeneSeek Genomic Profiler Bovine 100K, and GeneSeek Genomic Profiler Bovine HDv3 (150K). The genome positions of SNPs in different chips were unified according to the cattle reference genome assembly ARS-UCD 1.2 using CrossMap v0.5.2 [13] and then all the genotype data of all chips were imputed to the sequence level using a reference panel of the WGS data of 1059 Holstein cattle (average sequencing depth = 15.33×) from 1000 Bull Genome Project Run 8 [2], as elaborated in our previous study [14]. Based on the imputed data and the known SNPs and INDELs, we identified 21,975,591 SNPs and 1,983,871 INDELs. Then, we removed SNPs and INDELs with MAF less than 0.05 or with *p*-value < 1 × 10^−6^ for the Hardy–Weinberg Equilibrium test, resulting in 11,133,463 SNPs and 992,853 INDELs for the subsequent analysis.

Five milk production traits, including milk yield (MY), milk fat yield (FY), milk protein yield (PY), milk fat percentage (FP), and milk protein percentage (PP), were analyzed. De-regressed proofs (DRPs) derived from estimated breeding values (EBVs) using the method of Garrick et al. [15] were used as the pseudo-phenotype in the GWAS analysis. These EBVs were 305-day EBVs, which were obtained based on a multi-trait multi-lactation random regression test-day model.

### 2.2. GWAS Analysis

We first pruned the SNPs and INDELs with respect to LD using Plink v1.9 [16] with *r*^2^ = 0.2, respectively, resulting in 493,338 SNPs and 89,103 INDELs, which were used for GWAS analysis. For each SNP or INDEL, the following model was used for association analysis:

y=1μ+xβ+Za+e

where *y* is the vector of DRPs of one of the five traits, μ is the overall mean, β is the effect of the SNP or INDEL being analyzed, *x* is the vector of genotypes (coded as 0, 1, and 2, corresponding to the three genotypes), *a* is the vector of random polygenic effects with distribution of $N(0,G\sigma_{a}^{2})$, *Z* is the design matrix of *a*, *e* is the vector of random residuals with distribution of $N(0,I\sigma_{e}^{2})$, and *G* is the genomic relationship matrix built with the method of VanRaden [17].

The variance components ($\sigma_{a}^{2}$ and $\sigma_{e}^{2}$) in the model were estimated by restricted maximum likelihood (REML) method using the software GMAT v1.0 [18]. The Wald chi-squared test was used to examine the significance of the SNP or INDEL effect, and Bonferroni correction was used to account for multiple testing (threshold = 0.05/493,338 = 1.01 × 10^−7^ for SNP GWAS, or 0.05/89,103 = 5.61 × 10^−7^ for INDEL GWAS).

### 2.3. Conditional GWAS

For each significant INDEL, we calculated its LD (*r*^2^) with the nearby significant SNPs within 150 kb around it. If the *r*^2^ between the INDEL and at least one SNP was ≥0.2, we regarded the association of the INDEL being due to its strong LD with the SNP. Otherwise, we conducted conditional association analyses for the INDEL by fitting the most significant SNP within 150 kb around it in the model to test if the INDEL is independently associated with the trait. If there was no significant SNP within 150 kb around this INDEL, we took the nearest significant SNP within 1 Mb to perform the conditional association analysis. If there was no significant SNP within 1 Mb, we regarded this INDEL as independently associated with the trait.

### 2.4. Gene Annotation

For significant INDELs and SNPs, we performed gene annotation based on the cattle reference genome assembly ARS-UCS 1.2 (https://ftp.ensembl.org/pub/release-110/gtf/bos_taurus/, accessed on 12 January 2024). If the significant INDELs or SNPs did not overlap with any gene, we reported the 2 closest genes at either side of the them.

## 3. Results

### 3.1. SNP-Based GWAS

**Figure 1:**
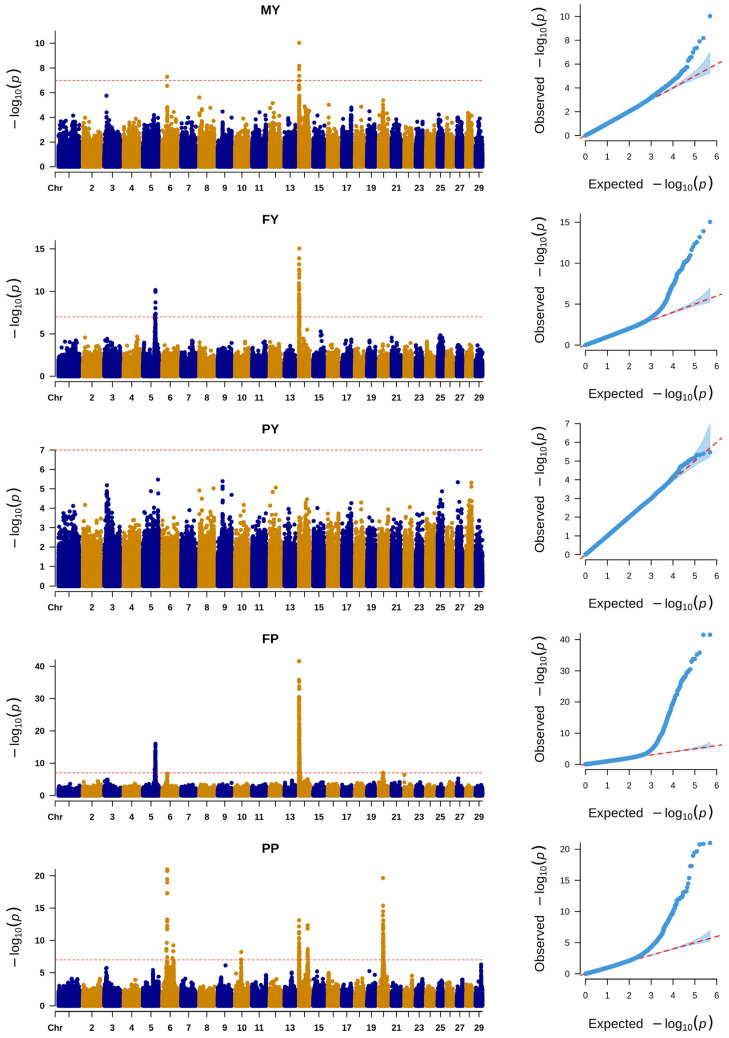
Manhattan and QQ plots of GWAS of SNPs for milk yield (MY), milk fat yield (FY), milk protein yield (PY), milk fat percentage (FP), and milk protein percentage (PP). The red straight line indicates the significant threshold of the *p*-value = 1.01 × 10^−7^.

The results of the SNP-based GWAS are illustrated in Figure 1. The significant SNPs for MY, FY, FP, and PP were 5, 57, 263, and 135, respectively. No significant SNPs for PY were identified. Some SNPs were significant for more than one trait and the number of unique significant SNPs was 369.

### 3.2. INDEL-Based GWAS

**Figure 2:**
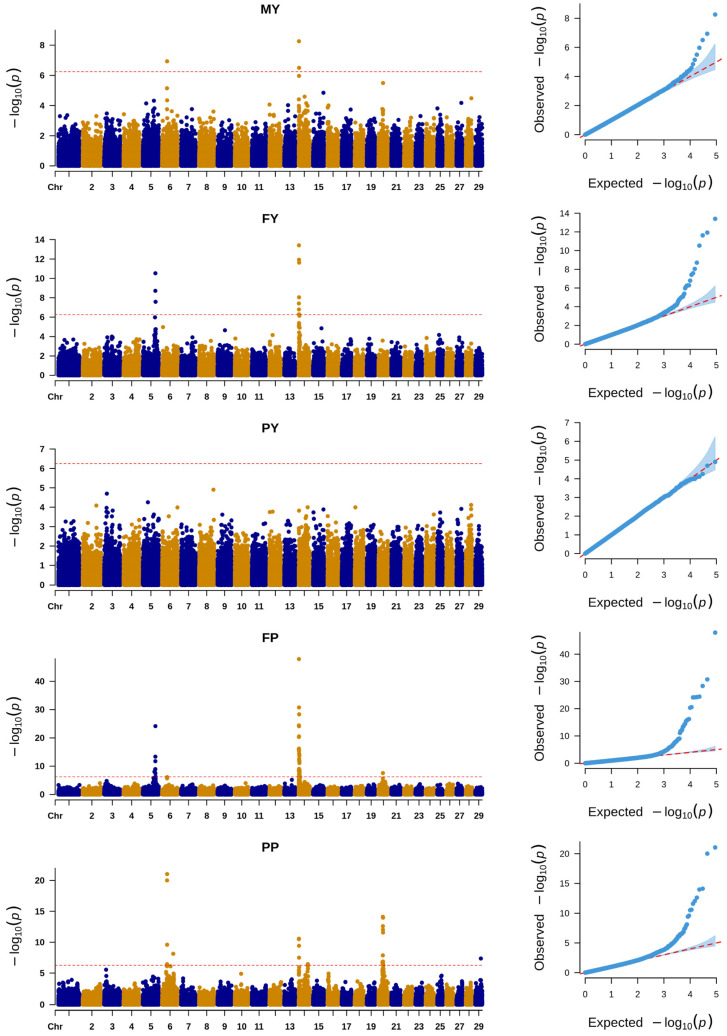
Manhattan and QQ plots of GWAS of INDELs for milk yield (MY), milk fat yield (FY), milk protein yield (PY), milk fat percentage (FP), and milk protein percentage (PP). The red straight line indicates the significant threshold of the *p*-value = 5.61 × 10^−7^.

The results of the INDEL-based GWAS are illustrated in Figure 2. The significant INDELs for MY, FY, FP, and PP were 3, 12, 46, and 22, respectively. Again, for PY, we did not identify significant INDELs. The number of unique significant INDELs was 58. Detailed information about these INDELs is given in Table S1.

### 3.3. Conditional Association Analysis

We first looked at the INDELs for which there were significant SNPs nearby within 150 kb. There were 38 such INDELs and their LD (*r*^2^) with the most significant nearby SNPs are shown in Table S2. A total of 34 of these INDELs were in strong LD (*r*^2^: 0.5–1) with at least one of the nearby significant SNPs. However, there were four INDELs (INDEL (Chr6:36865910), INDEL (Chr14:266639), INDEL (Chr14:1508606), and INDEL (Chr14:2195228)) which showed weak LD (*r*^2^ < 0.1) with nearby significant SNPs. In addition, there were 10 INDELs for which no significant SNPs within 150 kb were identified (Table S3), of which six had significant SNPs within 1 Mb and four had not. For each of the INDELs which had significant SNPs within 150 kb or 1 Mb (eight in total), we performed a conditional association analysis with respect to the most significant SNP within the 150 kb or the nearest significant SNP within 1 Mb. It turned out that five INDELs remained significant after adjusting the conditional SNPs (Table 1), among which INDEL (Chr14:920017) was significant for three traits, particularly for fat percentage with a *p* value of 3.22 × 10^−8^. These five INDELs and the four without significant SNPs within 1 Mb (Table 2) were considered independently associated with milk production traits.

## 4. Discussion

We identified 1,983,871 INDELs (992,853 after quality control) in the cattle genome from imputed sequence data of 6649 Holstein cows. This means that there is an INDEL about every 1.5 kb on average. Among them, 1,160,266 are deletions and 823,605 are insertions. This number is comparable with that reported in Hayes and Daetwyler [2] who identified 1,758,189 INDELs from sequence data of 2241 bulls of 61 Bos Taurus breeds in the frame of the 1000 Bull Genome Project.

We performed GWASs of INDELs for five milk production traits in the 6649 Holstein cows. To our knowledge, this is the first INDEL-based GWAS in dairy cattle. We identified 58 unique significant INDELs for one or multiple traits. The majority of these INDELs are in considerable LD with nearby significant SNPs and the annotated genes to these INDELs were also annotated with the relevant SNPs. Therefore, on the sequence level, most genetic variations captured by INDELs are also captured by SNPs because of the strong LD between INDELs and SNPs. However, there are still some INDELs which cause a small number of genetic variations (nearly) independently. Though conditional association analysis, we identified nine INDELs which showed independent associations, of which five had significant SNPs within 150 kb or 1 Mb nearby and four had no significant SNPs within 1 Mb. Of the nine INDELs, four are associated with FP (INDEL (Chr5:86639620), INDEL (Chr14:266639), INDEL (Chr14:920017), and INDEL (Chr20:31976989)), and five are associated with PP (INDEL (Chr6:36865910), INDEL (Chr6:36443645), INDEL (Chr14:67765289), INDEL (Chr20:28876195), and INDEL (Chr20:29002625)). Additionally, INDEL (Chr14:920017) is also associated with fat yield and milk yield. In the following, we summarize the annotated genes related to these INDELs and their functions and previously reported associations with milk production traits.

INDEL (Chr5:86639620) is an insertion, located ~8 kb upstream of the *SOX5* gene and ~463 kb downstream of the *TRNAG-CCC* gene. *SOX5* (SRY-box transcription factor 5) is a transcription factor and is involved in cell fate commitment, chondrocyte differentiation, and transcription by RNA polymerase II. Jiang et al. [19] and Tribout et al. [20] identified some SNPs within or close to *SOX5* which are significantly associated with FP. *TRNAG-CCC* (transfer RNA glycine (anticodon CCC)) is a transfer RNA gene. Transfer RNA are well known for their role in the essential link between the genetic code and amino acids and have been commonly considered housekeeping molecules that are intimately linked to cell proliferation and cell-cycle control. In humans, however, it is becoming increasingly clear that tRNAs are highly regulated, and that even small changes in their abundance or their nucleotide modification levels can have profound effects, leading to aberrant translation, changes in protein expression, and disease states [21].

INDEL (Chr14:266639) is a deletion, located in an intron of the protein-coding gene *ZNF16*. Reports of *ZNF16* (zinc finger protein 16) function are few and limited. Some studies suggested that *ZNF16* is involved in megakaryocytic and erythroid differentiation [22,23]. George and Diaz-Martinez revealed a novel role of *ZNF16* in rRNA transcription, suggesting that *ZNF16* may play a role in basic cellular processes [24]. Buitenhuis et al. [25] and Jiang et al. [19] reported several SNPs upstream of *ZNF16* which were significantly associated with FP in Holstein cattle, but the distances between these SNPs and *ZNF16* were greater than 1.4 Mb.

INDEL (Chr14:920017) is an insertion, located ~41 kb upstream of the *MIR2309* and ~16 kb downstream of the *EPPK1* gene. *MIR2309* is a microRNA gene with unknown function and no reports on its association with FP. *EPPK1* is a protein-coding gene which plays a role in the production of unsaturated fatty acids [26]. Very recently, Hosseinzadeh et al. [27] found that the expressions of *EPPK1* in the blood, lung, hypothalamus, and uterus were associated significantly with milk production traits based on a transcriptome-wide association study. They further identified that *EPPK1* is one of the four common significant genes between biological processes, cellular components, and molecular function based on GO analysis. This may be attributed to its effects on multiple traits (MY, FY, and FP).

INDEL (Chr20:31976989) is a deletion, located in an intron of the *GHR* gene. A large number of studies reported significant SNPs within or close to *GHR* for FP as well as many other traits in cattle (Cattle QTL Database, https://www.animalgenome.org/cgi-bin/QTLdb/BT/index, accessed on 20 January 2024). In this study, we also found several significant SNPs for FP within or close to GHR. After conditional analysis on the most significant SNP, this INDEL remained significant, suggesting it has extra effects on FP.

INDEL (Chr6:36865910) is an insertion, located within an intron of *LOC112447053*, a long non-coding RNA with unknown function with no reports on its association with milk production traits.

INDEL (Chr6:36443645) is an insertion, located ~77.4 kb upstream of *HERC6* and 5.5 kb downstream of *PPM1K*. *HERC6* is a member of the E3 ubiquitin protein ligase family containing HECT and RLD domains, while ubiquitin ligases play a role in milk protein synthesis [28]. A GWAS in Holstein cattle by Tribout et al. also identified an SNP within an intron of *HERC6* with a significant effect on PP [20]. *PPM1K* is a protein phosphatase. No report on its association with PP was found. However, some studies revealed that *PPM1K* could regulate branched chain amino acids and thus play a role in fat and protein synthesis [28].

INDEL (Chr14:67765289) is a deletion, located ~57 kb upstream of *SDC2* and ~128 kb downstream of *PTDSS1. SDC2* (Syndecan-2) is a member of the syndecan family. Syndecan-2 functions as an integral membrane protein and participates in cell proliferation, cell migration, and cell–matrix interactions via its receptor for extracellular matrix proteins. In humans, it has been proved that SDC2 is involved in tumor angiogenesis that facilitates tumor growth and metastasis [29]. In dairy cattle, Ning et al. [30] identified an intronic SNP of *SDC2* associated with PP. *PTDSS1* encodes phosphatidylserine synthase 1 (PSS1), one of two enzymes involved in the production of phosphatidylserine (PS). PS is the active substance of the cell membrane and plays an important role in the cell structure and cell signaling. Sousa et al. [31] identified missense mutations in *PTDSS1,* causing a gain-of-function effect associated with the regulatory dysfunction of PSS1. Although there is no report on the association of *PTDSS1* and milk protein traits, it could be a potential candidate gene for PP because of its function in PS synthesis.

INDEL (Chr20:28876195) (insertion) and INDEL (Chr20:29002625) (deletion) are both located upstream of *mir-10163* (87 kb and 214 kb, respectively) and downstream of *HCN1* (235 kb and 108 kb, respectively). *mir-10163* has numerous targets including retinoic acid receptor RXR-alpha (RXRA), which is involved in cell proliferation and apoptosis [32]. *HCN1* encodes a kind of membrane protein which can homodimerize or heterodimerize with other pore-forming subunits to form a potassium channel. In humans, *HCN1* has been proved to be involved in epilepsy diseases, including genetic generalized epilepsies, epilepsy with febrile seizures plus, epileptic encephalopathy, early infantile epileptic encephalopathy, catastrophic epilepsies, and so on [33]. In dairy cattle, Jiang et al. [34] reported several significant intronic SNPs within *HCN1* for PP.

## 5. Conclusions

In conclusion, we identified nine INDELs which were significantly and independently associated with one or multiple milk production traits. Among the genes relevant to these INDELs (harboring or close to the INDELs), some, i.e., *SOX5*, *ZNF16*, *GHR*, *HERC6*, *SDC2*, and *HCN1*, have been reported as associated genes for the corresponding traits based on SNP-based GWASs. The other genes, including *TRNAG-CCC*, *EPPK1*, *PPM1K*, *PTDSS1*, and *mir-10163,* could also be potential candidate genes for milk production traits according to their functions. Our findings indicate that INDEL-based GWASs could be valuable complements to SNP-based GWASs for milk production traits. However, it is essential to acknowledge our study limitations. Firstly, it should be noted that we focused on a few candidate genes related to the independently significant INDELs, but this does not mean that the other genes related to other significant INDELs are not important. Secondly, functional validations of these genes are required to confirm their causal effects on the studied traits.

## Figures and Tables

**Table 1:** Significant INDELs from the conditional association analyses.

INDEL	Conditional SNP	Distance	Traits	*p*-Value
INDEL (Chr6:36865910)	SNP (Chr6:36956973)	91,063	PP	3.73 × 10^−4^
INDEL (Chr6:36443645)	SNP (Chr6:36601824)	158,179	PP	1.67 × 10^−3^
INDEL (Chr14:266639)	SNP (Chr14:269574)	2935	FP	7.56 × 10^−4^
INDEL (Chr14:920017)	SNP (Chr14:512818)	407,199	MY	3.43 × 10^−3^
INDEL (Chr14:920017)	SNP (Chr14:1045680)	125,663	FY	1.57 × 10^−3^
INDEL (Chr14:920017)	SNP (Chr14:1045680)	125,663	FP	3.22 × 10^−8^
INDEL (Chr20:31976989)	SNP (Chr20:31273619)	703,370	FP	2.54 × 10^−3^

**Table 2:** INDELs without significant SNPs within 1 Mb.

Traits	INDELs		Nearest sig. SNPs		Distance
	ID	*p*-Value	ID	*p*-Value	
FP	INDEL (Chr5:86639620)	9.36 × 10^−9^	SNP (Chr5:87857269)	8.11 × 10^−9^	1,217,649
PP	INDEL (Chr14:67765289)	3.58 × 10^−7^	SNP (Chr14:65872284)	1.11 × 10^−8^	1,893,005
PP	INDEL (Chr20:28876195)	1.61 × 10^−7^	SNP (Chr20:30149443)	1.31 × 10^−14^	1,273,248
PP	INDEL (Chr20:29002625)	2.56 × 10^−7^	SNP (Chr20:30149443)	1.31 × 10^−14^	1,146,818

## Data Availability

The original contributions presented in the study are included in the article/Supplementary Material, further inquiries can be directed to the corresponding author.

## Supplementary Materials

The following supporting information can be downloaded at https://www.mdpi.com/article/10.3390/ani14243556/s1. Table S1: Significant INDELs associated with milk production traits. Table S2: INDELs with significant SNPs within 150 kb and the LDs between them. Table S3: INDELs without significant SNPs within 150 kb.
